# The Active Learning–GenAI Synergy Framework: Ethical Integration of Generative AI in EFL/ESL Writing in Resource-Constrained Contexts

**DOI:** 10.12688/f1000research.171435.2

**Published:** 2026-03-03

**Authors:** Shaista Rashid, Sadia Malik, Fatima Ghauri

**Affiliations:** 1 Linguistics and Translation, Prince Sultan University, Riyadh, Saudi Arabia; 2 Department of English, Bahauddin Zakariya University, Multan, Punjab, Pakistan

**Keywords:** Active Learning, Generative AI, EFL Writing, Cognitive Partnerships, Educational Technology, Academic Integrity, Higher Education

## Abstract

**Purpose:**

This mixed-methods action research study investigates the integration of ethically designed generative AI tools as catalysts for active learning in English as Foreign Language (EFL) writing instruction. The research is situated within resource-constrained higher education contexts and is grounded in Kolb’s experiential learning theory and active learning principles.

**Design/methodology:**

The study employed a quasi-experimental design involving 148 undergraduate students from Bahauddin Zakariya University, Pakistan. Over a 15-week intervention, an experimental group utilized AI tools (ChatGPT, Claude AI, Meta AI, and Canva) within a cognitive partnership model, while a control group received traditional teacher-centered instruction. Quantitative data on writing performance was supplemented by qualitative data from semi-structured interviews to capture student experiences and the development of ethical awareness.

**Findings:**

Quantitative results show the experimental group achieved statistically significant improvements in writing performance (Z = -6.325, p < .001) compared to modest gains in the control group (Z = -2.128, p = 0.033), with notable skill progression emerging after 6-8 weeks. Qualitative analysis revealed that AI tools successfully functioned as cognitive partners, metacognitive mirrors, and equity tools. A strong majority of participants (79.7%) expressed positive views about AI integration, with 86% indicating intentions for continued use.

**Originality/value:**

The paper provides a practical model for embedding AI tools in collaborative-learning settings while upholding academic integrity through a disciplined process of ethical reflection. The findings challenge the presumption that technology alone drives educational improvement, demonstrating instead that it is the pedagogical structures mediating AI interaction that determine an effective and ethical educational process.

## 1. Introduction

The rapid increase in the use of Artificial Intelligence (AI) in education has redefined the way pedagogy is conducted across disciplines, and the implications of the practice are significant in terms of language learning environments. On the one hand, in the case of English as a Foreign Language/Second Language (EFL/ESL) learning, students have fewer possibilities to have individualized learning and practice authentic communication, so AI opens up new opportunities regarding the dynamism in the process and individualization of the student. Such transformation is particularly topical in terms of the active learning theory that prioritizes the importance of engagement, critical thought, and jointly constructed knowledge as opposed to passive reception of information ( Prince, 2004; Freeman et al., 2014).

In comparison with lecture-dominated pedagogies prevalent in many EFL/ESL teaching settings, especially those plagued by poor resources, active learning can be described as any teaching technique that involves the students in the learning process. Bonwell and Eison (1991) argue that active learning requires participation in meaningful activities accompanied by reflection, promoting deeper engagement and stronger outcomes. In writing instruction, these principles align with process-oriented approaches, transforming composition from a product-driven task into a recursive, collaborative, and reflective practice ( Flower & Hayes, 1981; Graham & Perin, 2007).

The convergence of AI technologies and active learning offers transformative potential for EFL/ESL writing instruction ( Imran & Almusharraf, 2023). Generative AI tools such as ChatGPT, Claude AI, Meta AI, and Canva can function as cognitive partners, enabling interaction, personalized feedback, collaborative idea generation, and metacognitive reflection ( Zawacki-Richter et al., 2019; Chen et al., 2020; Yang et al., 2022). These tools support Kolb’s (1984) experiential learning cycle, allowing learners to draft with AI (concrete experience), critique AI outputs (reflective observation), extract principles (abstract conceptualization), and revise iteratively (active experimentation).

Integrating AI in pedagogy requires more than technological adoption; it demands reconceptualizing learning as a partnership between learners, AI, and pedagogy ( Luckin, 2017; Imran et al., 2024). In this model, AI works not in opposition to cognition but as a catalyst of active learning, reducing classroom limitations, including large student numbers, little feedback, and a lack of collaboration possibilities ( Godwin-Jones, 2019; Holmes et al., 2019).

The Pakistani higher education environment depicts all these restrictions and possibilities. The history of writing instruction in Pakistani universities has been rich in grammar precision and fixed modes of expression instead of rhetorical elegance and thinking ( Mahmood et al., 2020). Students need help in planning, organizing, and revising their work so that they can write coherently. These are coupled with teacher-oriented pedagogies and huge classes that inhibit advice and restrain collaboration ( Rashid & Malik, 2025).

Within this context, ethically situated AI integration can make writing classrooms active learning epistemologies. AI tools also offer proximal feedback, guidance, and means to collaborate with others and thus participate in spite of systemic constraints ( Blikstein, 2016; Popenici & Kerr, 2017; Renz et al., 2020). The idea of positioning students as collaborators with AI instead of passive inculcators can help avert critical thinking, metacognition, and learner autonomy, the hallmarks of effective active learning ( Prince, 2004; Freeman et al., 2014).

The ethical aspect is primary. Active learning is focused on agency, critical assessment, and genuine involvement, which can suffer dilution as a result of AI-established shortcuts ( Anderson & Rainie, 2023; HEC, 2023). Ethical frameworks, in turn, must emerge to help promote academic integrity, critical evaluation of AI output, and maintain an emphasis on human development optimism as opposed to technological dependence.

The research idea is to explore ways in how ethically scaffolded generative AI tools are able to accelerate active learning in EFL writing courses, specifically in resource-limited Pakistani higher education. By exploring AI-based writing pedagogy through the experiential learning cycle of Kolb (1984) and Bonwell and Eison (1991), and active learning ideas ( Prince, 2004), we can relate how AI drives active processes of learning, interaction, and reflection to improve writing proficiency and digital literacies.

The paper has thus revealed how AI can be adopted in active learning as a cognitive companion instead of an active involvement as a barrier to implementing traditional pedagogy or delivering a learning space free of discrimination. Hence, this study attempts to answer three questions:

**RQ1**: How does generative AI integration within active learning environments enhance EFL/ESL writing outcomes compared to conventional teacher-centered instruction in resource-constrained contexts?

**RQ2**: What ethical integration strategies promote critical engagement and reflective practice when EFL/ESL learners collaborate with generative AI tools during writing tasks?

**RQ3**: How do ethically scaffolded AI tools function as cognitive partners to support personalized active learning experiences and address systemic constraints in large EFL/ESL writing classes?

### 1.1 Significance of the study

This research makes key contributions to AI-enhanced active learning in EFL/ESL contexts. Theoretically, it develops an Active Learning–GenAI Synergy Framework that positions AI tools as cognitive partners, contributing to theories of human–AI collaboration ( Luckin, 2017; Holmes et al., 2019). Pedagogically, it demonstrates how ethically integrated AI can shift writing instruction from teacher-centered to dynamic active learning, offering evidence for student-centered approaches in resource-limited contexts ( Prince, 2004; Freeman et al., 2014). Practically, it provides scalable solutions for large EFL/ESL writing classes in Pakistani higher education, informing curriculum development, instructor training, and AI integration policies.

## 2. Literature review

### 2.1 Active learning theory: Foundations and principles

The active learning process is inherently a challenge to the traditional transmission model, which inculcates a student as an active knowledge builder, as opposed to a receiver of the knowledge. The working definition of student-centered learning by Bonwell and Eison (1991) refers to any instructional approach that involves students in the process of learning, stressing that students should do more than merely being asked to listen; they need to read, write, talk, or be asked to solve problems. The idea of this definition focused on interaction, participation, and thinking, not on the consumption of content.

Prince (2004) extended this framework by emphasizing the metacognitive dimension, noting that active learning requires students to engage in “meaningful learning activities and think about what they are doing” (p. 223). This metacognitive emphasis becomes particularly relevant in AI-mediated contexts where tools can facilitate reflective practice through feedback and revision cycles.

Freeman et al.’s (2014) comprehensive meta-analysis of 225 studies provided compelling empirical support, revealing that active learning increased student performance by 6% and reduced failure rates by 36% compared to traditional lecturing. Their conclusion that active learning represents “the preferred, evidence-based choice for instructors” (p. 8410) has been replicated across disciplines, establishing active learning’s superiority over instructor-centered methods and providing the theoretical foundation for AI-enhanced pedagogical approaches.

### 2.2 Theoretical frameworks of active learning

Active learning theory draws from three foundational frameworks that inform AI integration in educational contexts. Social constructivism emphasizes Vygotsky’s (1978) Zone of Proximal Development (ZPD), where AI tools function as “more knowledgeable others” providing scaffolding through immediate feedback and collaborative dialogue. Johnson and Johnson (2009) extend this through collaborative learning theory, demonstrating that learning is enhanced when students work toward shared goals—processes readily facilitated through AI-mediated peer collaboration.

The main theory of AIs-enhanced active learning cycles is experiential learning theory. The four-step model offered by Kolb (1984), concrete experience, reflective observation, abstract conceptualization, and active experimentation, corresponds directly to collaborations with AI-writing: students take concrete steps in writing with AI assistance, contemplate the results produced by AIs, theoretically abstract writing concepts based on the suggestions provided by the AI, and implement experimental approaches to revision.

### 2.3 Active learning in writing instruction

Collaborative and back and forth writing pedagogy inherently lends itself to active learning. In their meta-analysis, Graham and Perin (2007) also found that active learning strategies that show significant improvement in writing are collaborative writing (effect size = 0.75), strategy instruction (effect size = 0.82), and peer assistance (effect size = 0.89). These results indicate the usefulness of AI integration, which can provide more teamwork experiences and direction.

Flower and Hayes (1981) redefined composition as a cycle of planning, translating, and reviewing-with metacognitive activity and reflection on revision. AI-based solutions can facilitate every step with idea generation, vocabulary proposals, and automatic feedback. Bereiter and Scardamalia (1987) differentiate between the knowledge-transforming process of writing, which presupposes an active treatment of content and development of arguments, and the knowledge-telling paradigm in which AI can become an aid to critical reflection and perspective analysis.

### 2.4 AI applications in writing instruction: Evolution and effectiveness

As writing applications have developed beyond rudimentary grammar checkers, this has also meant a shift toward active learning pedagogies and a greater emphasis on student agency and collaboration ( Nazari et al., 2021; Imran & Almusharraf, 2024).

**2.4.1 Early systems and feedback tools**

Early experiments were on automated feedback and grammatical markup. Purcell et al. (2013) discovered that Google Docs supported enhanced writing with the help of scaffolding and collaborative writing features. Nonetheless, tools demanded organized instructional schemes that supported critical participation, which was the core of active learning theory ( Nobles & Paganucci, 2015).

**2.4.2 Advanced evaluation systems**

Sophisticated automated writing evaluation (AWE) technology was an important step forward. Fitria (2021) has documented that AI-based applications such as Grammarly fostered metacognitive awareness and reflective practice through facilitating critical self-assessment. Chang et al. (2021) reported statistically significant gains when AI tools gave instantaneous, domain-specific feedback in addition to increasing the independence of the learners-commonly referred to as active learning.

**2.4.3 Contemporary generative AI integration**

Recent generative AI research provides new active-learning opportunities. Su et al. (2023) showed how argumentative writing is done through collaboration of students with the AI partner, ChatGPT, as a cognitive partner to support collaborative learning and knowledge construction. Song and Song (2023) identified increases in coherence, order, and drive when AI was incorporated into structured frameworks, with a call to employment in moderation to encourage agency over reliance. Huang et al. (2023) contrasted AI-aided and conventional settings. Under the AI setting, where students received personalized prompts and guidance to enhance revision tasks, academic performance was more effective, and engagement was higher than in the traditional setting.

### 2.5 Ethical AI integration and active learning

The combination of AI technology and active learning pedagogy proposes significant ethical issues that need to be discussed to ensure that the educational outcomes of the integration of technology are productive, not destructive. Active learning theory is based on the principles of student choice, critical thinking, and genuine engagement, which may be negatively affected by the misuse of AI tools.

Regarding ethical use of AI in higher education, policy guidelines were created by UNESCO (2021) and the Higher Education Commission of Pakistan (2023), which were outlined not only as essentials to preserve academic integrity but also to take advantage of the technological affordances. These rules reflect the active learning theory, encouraging responsibility, critical thinking, and reflection of the students engaged in learning conditions provided by an AI system.

In the case of the ethical application of AI in higher education, the Higher Education Commission of Pakistan developed institutional policies (2023) that were described as necessary to ensure the absence of academic dishonesty and to utilize technological capabilities. These instructions are consistent with active learning theory and foster responsibility, critical thinking, and reflection among students in the learning conditions an AI system presents.

The framework is in line with the international standards: educational AI is classified as a high-risk area that needs transparency, human control, and risk management ( EU AI Act, 2024); the recommendations on generative AI in education and research (2023) by UNESCO focus on critical digital literacy, academic integrity, and fair access; the Safe AI in Education Manifesto includes the focus on preserving human agency and avoiding dependency ( UNESCO, 2023). These convergent principles were used in designing protocols.

In the case of the ethical application of AI in higher education, the Higher Education Commission of Pakistan developed institutional policies (2023) that were described as necessary to ensure the absence of academic dishonesty and to utilize technological capabilities. These instructions are consistent with active learning theory and foster responsibility, critical thinking, and reflection among students in the learning conditions an AI system presents.

### 2.6 Research gaps and theoretical synthesis

Although research on AI in writing instruction is growing, there are noticeable gaps in how generative AI tools can be ethically integrated into active learning approaches, especially in EFL/ESL settings where resources are limited. The current state of research on AI effectiveness focuses on pedagogical curriculum, supporting student agency, critical thinking, and group learning insufficiently. Most studies have been carried out in high-resource learning environments and do not provide many insights into how AI tools can tackle systemic issues like excessive classroom sizes, poor quality teacher feedback, and the availability of resources to provide personalized learning. The present study fills these gaps by exploring how ethically scaffolded generative AI tools can be used to promote active learning in EFL/ESL writing instruction.

## 3. Research methodology

### 3.1 Research design and theoretical alignment

The pragmatic mixed-methods action research design is employed by combining quantitative performance measures and a qualitative phenomenological evaluation through which the full comprehension of AI-mediated active learning experiences was understood. The action research framework’s emphasis on cyclical reflection, pedagogical innovation, and collaborative inquiry aligns with both active learning theory and ethical AI integration practices ( Stringer, 2014; McNiff & Whitehead, 2011).

The design entails pre-test/post-test experimental and control group comparisons supplemented with semi-structured interviews, where methodological triangulation explored the relationship between the AI-enhanced active learning performance (quantitative measure) and the quality of student engagement coupled with ethical awareness development (qualitative measure). Action research aspect facilitated ongoing pedagogical upgrades over the course of intervention (15 weeks) and made real-time alterations to AI integration plans through student feedback- in keeping with the iterative, reflective qualities of active learning and ethical AI usage.

### 3.2 Theoretical framework integration: Active learning theory and Kolb’s experiential learning cycle

The methodological design is grounded in Kolb’s Experiential Learning Theory, which posits that “learning is a continuous, cyclical process involving four key stages: Concrete Experience, Reflective Observation, Abstract Conceptualization, and Active Experimentation” ( Kolb, 1984). This theory states that “learning is the process whereby knowledge is created through the transformation of experience. Knowledge results from the combination of grasping and transforming experience”.

The AI-enhanced writing tasks were systematically designed to facilitate each stage of Kolb’s experiential learning cycle:

1. **Concrete Experience (CE)**: Students engaged in hands-on writing activities using AI tools (ChatGPT, Claude AI, Meta AI, Canva) to draft, revise, and create multimodal compositions
2. **Reflective Observation (RO)**: Weekly ethical reflection prompts encouraged students to critically analyze AI outputs, evaluate their own contributions, and observe the collaborative writing process
3. **Abstract Conceptualization (AC)**: Students developed an understanding of writing principles, ethical AI use, and metacognitive strategies through guided reflection and peer discussion
4. **Active Experimentation (AE)**: Students applied insights from reflection to subsequent writing tasks, experimenting with different AI collaboration strategies and testing ethical integration approaches

**Active learning principles integration**

The methodology incorporated core active learning principles identified by Prince (2004) and Freeman et al. (2014):

• **Student Engagement**: AI tools facilitated interactive writing processes that required continuous student participation and decision-making
• **Collaborative Learning**: Peer review activities were enhanced through AI-assisted feedback and structured group reflection sessions
• **Higher-Order Thinking**: Ethical reflection prompts (See Appendix 3) promoted critical evaluation, synthesis, and evaluation of AI-generated content
• **Immediate Feedback**: AI tools provided real-time responses that enabled rapid iteration and experiential learning cycles
• **Student Agency**: Learners maintained control over AI collaboration, making autonomous decisions about when and how to integrate AI suggestions

**Technology Acceptance Model (TAM) Integration**

Furthermore, Davis’s (1989) Technology Acceptance Model provided the theoretical framework (See Appendix 5) for understanding student acceptance and utilization of AI tools within active learning contexts. The study investigated four key TAM constructs within the context of cognitive partnerships:

• **Perceived Usefulness (PU)**: The extent to which students believed AI tools enhanced their writing performance and learning outcomes
• **Perceived Ease of Use (PEOU)**: The degree to which students found AI collaboration effortless and intuitive
• **Attitude Toward Use (ATU)**: Students’ overall evaluative responses to AI-mediated active learning experiences
• **Behavioral Intention to Use (BITU)**: Students’ commitment to continued AI collaboration in future academic contexts

### 3.3 Participants and sampling strategy

The study employed convenience sampling to recruit 148 undergraduate students from Bahauddin Zakariya University, Multan, Pakistan. Participants were naturally occurring intact classes from two academic programs: the experimental group (Digital Marketing students) participated in AI-mediated active learning experiences, while the control group (BBA students) received conventional textbook-based writing instruction, ensuring ecological validity while maintaining practical feasibility within the resource-constrained context. The demographics of participants are given in Table 1.

**Table 1.** Participant demographics and group assignment.

Group	Program	Male students	Female students	Total	Active learning approach
Experimental	Digital Marketing	36	38	74	AI-Mediated Active Learning
Control	BBA	46	28	74	Traditional Instruction

The quasi-experimental design enabled receiving an opportunity to compare AI-mediated active learning to a conventional pedagogy based on teacher-centered education without any harm to ethics in the context of education equity. However, all results are of an exploratory nature due to the core confounded comparison because experimental and control groups are intact classes from different degree programmes, threatening internal validity. Pre-intervention assessment confirmed group comparability. Mann-Whitney U tests showed no significant differences in pre-test writing scores (Experimental: M rank = 72.45, SD = 8.32; Control: M_rank = 76.55, SD = 8.67; U = 2,561, p = .532). Both programs required equivalent English prerequisites (IELTS 5.5), with no differences in prior English grades (t = 0.58, p = .564). Demographics were comparable: gender distribution (χ ^2^ = 1.89, p = .169) and age (t = 0.73, p = .468). Both groups received instruction from qualified faculty (MA/MPhil in English, 6-8 years of experience) using standardized assessment rubrics, ensuring instructional consistency despite intact class design.

### 3.4 Instructional intervention design: AI-mediated active learning framework

The design of the instructional intervention in the experimental group involved an Active Learning-GenAI Synergy Framework that presented the AI tools as learning partners in collaborative opportunities. The three important roles that AI played under this intervention transformed conventional writing instruction in the following ways:

a. **AI as Cognitive Partner:** Tools were used to facilitate brainstorming, argumentation, and the ability to write multiple media, allowing the recreation of the writing process of writing as done in professional life
b. **AI as Metacognitive Mirror:** Features reflection tasks to direct AI criticism to enhance critical authorial self-assessment
c. **AI as Equity Tool**: AI provided personalized scaffolding and immediate feedback to address individual learning needs within large class constraints
d. **Tool Specifications & Access:** ChatGPT, Claude AI, Meta AI, Canva, all accessed via free institutional accounts.
e. **Permitted uses:** brainstorming, structural feedback, grammar checking, revision suggestions.
f. **Forbidden:** verbatim copying, unmodified AI text submission, and complete task delegation.

**Weekly active learning structure**

Each week followed a structured active learning cycle that integrated AI collaboration with experiential learning principles elaborated in Table 2.

**Table 2.** Weekly active learning cycle structure.

Kolb's stage	AI Integration Activity	Active Learning Component	Sample Prompt Example
Concrete Experience	AI-assisted writing task completion	Hands-on drafting and composition	Help structure formal letter: opening, body, closing
Reflective Observation	Ethical reflection prompt response	Critical evaluation of AI collaboration	What AI suggestions did I modify and why
Abstract Conceptualization	Peer discussion of AI insights	Collaborative knowledge construction	How did AI help understand coherence?
Active Experimentation	Revision based on reflection/discussion	Independent application of insights	Apply insights to revise draft

The instructional intervention spanned 15 weeks (See Appendix 2), from January 2025 to June 2025. The experimental group engaged with various AI tools, including ChatGPT, Claude AI, Meta AI, and Canva, while the control group continued with textbook-driven lessons focused on grammar drills, sample formats, and teacher explanations.

Each week in the experimental group was dedicated to a specific writing genre or task, with AI tools introduced as assistive scaffolds. These tools were not intended to replace human effort, but to guide students through revision, coherence building, vocabulary selection, and formatting tasks listed in Table 3.

**Table 3.** Weekly writing themes.

Week	Writing focus
Week 1-2	Informal Writing: Letters and Applications
Week 3-4	Formal Writing: Letters and Applications
Week 5-6	Prompt-Based Writing
Week 7-8	Cover Letter Writing
Week 9-10	CV Writing
Week 11-12	Business Proposal Writing
Week 13-14	Collaborative & Peer Writing Tasks
Week 15	Final Writing Task & Reflection

**Weekly Sequence:** (1) Genre introduction (15 min); (2) Prompt demonstration (10 min); (3) AI-assisted writing (60 min); (4) Strategy sharing (20 min); (5) Ethical reflection (20 min); (6) Submission with AI disclosure.

### 3.5 Ethical integration protocol

The study implemented a comprehensive Ethical Integration Protocol designed to ensure that AI collaboration promoted rather than undermined active learning principles:

This paper followed an elaborate Ethical Integration Protocol in accordance with the EU AI Act ( European Commission, 2024), UNESCO principles ( UNESCO, 2023), and Safe AI principles:

a. **Weekly Ethical Reflection Prompts:** Five-question protocol: (1) What AI tools were used and what? (2) What recommendations were accepted/declined and why? (3) Percentage difference between original and AI contribution? (4) Authentic voice maintained? (5) How did AI enhance learning? Required weekly submission.
b. **Academic Integrity Frameworks:** The problem of plagiarism prevention practices such as citation process, the distinction between AI support of the human mind versus AI or the substitution of the human mind have been discussed and dealt with regularly. It was explained to students that AI should not be perceived as cash generators but partners in the writing that cannot and must not be replaced.
c. **Crucial Digital Literacy Education:** Activities to challenge the quality of the AI output, detect the different biases, and come up with responsible practices of AI collaboration in academics.
d. **AI Tools and Pedagogical Integration:** Table 4 explains how different AI tools were integrated with principles of Active learning.

**Table 4.** AI tool integration and active learning applications.

AI tool	Primary function	Active learning integration	Kolb’s cycle stage
ChatGPT	Idea generation and dialogue	Interactive brainstorming sessions	Concrete Experience
Claude AI	Content structuring and coherence	Collaborative outline development	Abstract Conceptualization
Meta AI	Grammar and language enhancement	Peer-assisted revision activities	Active Experimentation
Canva	Multimodal composition	Creative collaborative projects	Concrete Experience

**Table 5.** GenAI risk register.

Risk	Mitigation
Academic dishonesty	Weekly disclosure; integrity agreements
Cognitive dependency	Mandatory original contribution analysis
Bias amplification	Critical evaluation training
Data privacy	Prohibition on personal data input
Inequitable access	Free accounts; lab access

### 3.6 Data collection methods and instruments

**Quantitative data collection**

• **Pre/Post Writing Assessments:** 100-point analytic rubric (See Appendix 6) assessed Coherence (25%), Organization (25%), Grammar (25%), Critical Thinking (25%) across four levels: Excellent (85-100), Good (70-84), Satisfactory (55-69), Needs Improvement (<55). Two trained raters scored blindly; inter-rater reliability κ = 0.84; discrepancies >10 points were adjudicated.
• **Ethical awareness** is defined as: (1) procedural knowledge (assistance vs. misconduct boundaries), (2) critical evaluation (assessing AI quality/bias), (3) metacognitive regulation (monitoring reliance).

Ethical Awareness: Coded reflection quality (1 = descriptive to 4 = metacognitive), AI disclosure accuracy, interview analysis. Progression: M = 1.8 (Week 1) to M = 3.4 (Week 15). No missing data. See complete rubric (Appendices-3).

**Weekly Writing Performance Tracking:** Six intermediate writing assessments provided a timeline on how students improved over the course of the intervention as well as the periods when students were making significant improvements towards the goal.

**Qualitative data collection**

Semi-Structured Interviews (See Appendix 4) were conducted through purposive sampling with eight participants, showing differing performance levels (3 high, 3 medium, 2 low achievers), gender-balanced (4F, 4M). Individual 30-45 min interviews explored cognitive partnership, ethical reflection, metacognitive development, active learning, and technology acceptance. Audio-recorded, transcribed verbatim, accuracy-checked. See full protocol in the Appendices-4.

### 3.7 Ethical considerations and safeguards

The study maintained rigorous ethical standards aligned with both research ethics and AI integration ethics by seeking approval from the Bahauddin Zakariya University’s Research Ethical Committee (Ref No. 28/UREC/2024 dated 16-10-2024) in compliance with the Declaration of Helsinki. Written Informed Consent was obtained (See Appendix 1) and all participants received comprehensive information about the study purposes, procedures, and their rights of voluntary participation, confidentiality, and participant anonymity. Educational Equity was ensured for the Control group that received access to AI integration training following study completion. For AI Ethics Integration, clear guidelines are distinguished between AI assistance and academic misconduct. Privacy Protection was ensured as no personal data was input into AI systems. Students disclosed AI use in all academic submissions as Transparency Requirements. Ethical reflection activities promoted responsible AI collaboration for critical engagement.

### 3.8 Data analysis framework

Analysis was quantitative based on Descriptive Statistics (Mean scores, standard deviations, and performance distributions of all writing assessments) and Inferential Testing (Within-group pre/post comparisons of non-parametric data) using Wilcoxon Signed-Rank Tests and Mann-Whitney U Tests (Between-group comparisons of experimental and control conditions), and Gender Analysis (Separate analyses of the varying effects of all demographic groups). Magnitudes that were given as rank-biserial correlation (r = Z/S N): small 0.30 or less, medium 0.30-0.49, large 0.50 or more. The Type I error was controlled by using Bonferroni correction on intermediate tests (0 = .008).

Six phases used by Braun and Clarke (2006): familiarization, coding (65 initial codes), theme generation, review, definition (five final themes), reporting. Two independent researchers coded 25 percent (κ = 0.82); the differences were resolved by discussion.

## 4. Findings

### 4.1 Active learning environment effectiveness: Quantitative outcomes

The quantitative analysis reveals significant evidence supporting the effectiveness of AI-mediated active learning environments compared to conventional teacher-centered instruction. These findings demonstrate how cognitive partnerships with AI tools facilitated measurable improvements in writing performance while promoting the student engagement and metacognitive development characteristic of active learning experiences.

**4.1.1 Overall writing performance: Active learning vs. traditional instruction**

The comparison between AI-mediated active learning (experimental group) and traditional instruction (control group) provides compelling evidence for the superior effectiveness of cognitive partnership approaches in EFL writing development given in Table 6.

**Table 6.** Pre- and post-intervention writing performance comparison.

Group	Test	*N*	Mean Rank	*Z*	*P*	Effect Size ( *r*)	Interpretation
AI-Active Learning (Experimental)	Pre-Test	74	63.47	−6.325	<.001	0.68	Highly significant improvement; large effect
	Post-Test	74	85.53				
Traditional Instruction (Control)	Pre-Test	74	73.12	−2.128	.033	0.23	Moderate improvement; small effect
	Post-Test	74	75.88				

The AI group showed great improvement with a large effect size (Z = -6.325, p < .001, r = 0.68), whereas the traditional instruction showed small improvement (Z = -2.128, p = .033, r = 0.23), indicating that cognitive partnerships led to better writing development.

**4.1.2 Progressive skill development through active learning cycles**

The analysis of writing performance across six intermediate assessments ( Table 7) reveals how cognitive partnerships with AI tools facilitated progressive skill development through repeated experiential learning cycles, consistent with Kolb’s (1984) theory of continuous learning improvement.

**Table 7.** Progressive writing performance across active learning cycles.

Writing Assessment	AI-Active Learning Mean Rank	Traditional Instruction Mean Rank	*P*	Effect Size ( *r*)	Bonferroni (α = .008)	Interpretation
Test 2 (Week 3–4)	78.35	70.65	.243	0.15	NS	Initial partnership formation
Test 3 (Week 5–6)	80.00	69.00	.069	0.24	NS	Borderline significance
Test 4 (Week 7–8)	79.15	69.85	.149	0.18	NS	Partnership consolidation
Test 5 (Week 9–10)	88.00	61.00	<.001	0.72	Sig	Partnership mastery; large effect
Test 6 (Week 11–12)	82.42	66.58	.001	0.58	Sig	Sustained effectiveness; large effect

The results show that there was an evident trend of progression in which the benefits of AI-mediated active learning became more prominent with time. There were marked differences at Test 5 (p < .001), so it appears that cognitive partnerships take time before they yield any meaningful benefit. This tendency contributes to the theory of experiential learning provided by Kolb that underlines the idea that learning becomes stronger with the number of cycles of experience, reflection, conceptualization, and experimentation.

Bonferroni correction (= .008) only confirms Tests 5-6 as significant with large effects (r = 0.72, r = 0.58); Tests 2-4 had small non-significant effects (r = 0.15-0.24), showing advantages occurred after 6-8 weeks without multiple testing inflation.

**4.1.3 Gender-differentiated active learning engagement**

Analysis of gender differences within the AI-mediated active learning environment ( Table 8) reveals differential engagement patterns, providing insights into how cognitive partnerships develop across demographic groups.

**Table 8.** Gender-based active learning engagement (AI-enhanced group).

Gender	*N*	Mean Rank	*Z*	*P*	Effect Size ( *r*)	Interpretation
Female students	38	41.53	−4.257	<.001	0.69	Highly significant improvement; large effect
Male students	36	33.25	−2.574	.010	0.43	Significant improvement; medium effect

Female students in the AI-mediated active learning environment demonstrated 19% higher engagement levels and a more significant improvement (p < .001) compared to male students (p = .010). Qualitative data suggests this difference may reflect greater responsiveness to the reflective observation and metacognitive awareness components of the experiential learning cycle, indicating that female students may engage more deeply with the ethical reflection prompts and collaborative peer activities that characterize active learning environments.

Notably, in the traditional instruction environment, the pattern reversed, with male students showing significant improvement while female students’ gains were not statistically significant ( Table 9). This contrast suggests that AI-mediated active learning environments may be particularly beneficial for female EFL learners, potentially because these environments emphasize collaborative reflection, peer interaction, and metacognitive awareness—learning approaches that align with documented female preferences for collaborative and reflective pedagogies.

**Table 9.** Gender comparison - Traditional instruction control group.

Gender	*N*	*Z*	*P*	Effect Size ( *r*)	Interpretation
Male students	46	−4.875	<.001	0.72	Significant improvement; large effect
Female students	28	−1.862	.062	0.35	Not statistically significant; medium effect

### 4.2 Cognitive partnership development: Qualitative insights

Semi-structured interviews with eight volunteer students from the AI-mediated active learning group provide rich insights into how cognitive partnerships developed and functioned within the experiential learning framework. These qualitative findings illuminate the mechanisms through which AI tools served as cognitive partners, metacognitive mirrors, and equity tools.

**4.2.1 AI as cognitive partner: Collaborative knowledge construction**

Students consistently described their relationships with AI tools in terms of collaborative partnership rather than tool usage, indicating successful development of the cognitive partnership model. Interview participants emphasized how AI tools supported active knowledge construction through interactive dialogue and collaborative problem-solving.

**Student Voice - Collaborative Idea Generation**: *“AI tools have assisted me a lot in writing. ChatGPT provided me with relevant arguments that helped me to improve my sentence structure, but I had to think critically about which ideas fit my own perspective.”*

This response demonstrates how students engaged in the Abstract Conceptualization stage of Kolb’s cycle, using AI input as a foundation for developing their own understanding rather than passively accepting generated content.

**Student Voice - Interactive Revision Process**: *“Claude AI was better for long paragraphs, and it gave me coherent ideas. But I learned to combine its suggestions with my own thoughts to create something that was truly mine.”*

This reflection illustrates Active Experimentation, where students tested different approaches to integrating AI suggestions within their own writing development process, demonstrating the experimental and iterative nature of effective active learning.

**4.2.2 AI as metacognitive mirror: Reflective awareness development**

The weekly ethical reflection prompts successfully promoted metacognitive awareness and reflective observation—key components of both active learning and cognitive partnership development. Students reported enhanced ability to evaluate their own learning processes and AI collaboration strategies.

**Student Voice - Metacognitive Development**: *“The weekly reflection prompts made me think about how I was using AI. I started to notice when I was depending too much on AI suggestions versus when I was using them to enhance my own ideas.”*

This response demonstrates the development of metacognitive awareness that enabled students to regulate their own learning and maintain agency within the cognitive partnership. Such self-awareness is essential for effective active learning and prevents the passive dependence that could undermine educational goals.

**Student Voice - Ethical Integration Success**: *“I avoided copying full paragraphs from AI. I learned to rewrite and reflect. The ethical prompts aided me in appreciating the distinction between AI help and AI substitution of my way of thinking.”*

This reflection demonstrates how the Ethical Integration Protocol was effective in facilitating responsible teamwork on AI that did not compromise academic integrity, while utilizing technological affordances, as one of the major goals of the active learning framework.

**4.2.3 AI as equity tool: Addressing resource constraints**

Students highlighted the ways AI tools solved age-old resource constraints in bigger EFL/ESL classes by providing individual learning experiences that would be otherwise unattainable in resource-poor settings.

**Student Voice - Personalized Learning Access:** *“Experiences with Canva to write proposals helped me to figure out how to structure information. Personal attention is something we do not usually get in large classes, but AI tools provided me individual feedback anytime it was necessary.”*

This is their answer, which shows the AI tools were equity tools that provided access to individualized instruction and real-time feedback on a massive scale and overcame the systemic barriers that generally limit active learning despite favorable evidence on the value of active learning.

**Student Voice - Increased Confidence and Agency:** “ *This has added confidence in writing emails and reports. I feel more confident about working independently, yet still there is support when I need.”*

This reflection demonstrates how cognitive partnerships facilitated self-efficacy and learner autonomy as the central by-products of active learning practices that help a student become a lifelong learner and ultimately a professional.

### 4.3 Technology acceptance within active learning contexts

Qualitative Analysis of interviews through the Technology Acceptance Model (TAM) framework reveals high levels of student acceptance of AI tools within active learning environments, with acceptance levels directly correlated to the degree of active engagement and cognitive partnership development.

**4.3.1 Perceived usefulness in active learning contexts**

The majority of participants expressed positive views about AI integration within active learning environments, with most of the experimental group students rating AI tools as “very effective” or “extremely effective” for developing language skills through active collaboration rather than passive consumption.

Students’ perceived usefulness was specifically tied to AI tools’ capacity to facilitate active learning processes:

• Interactive idea generation during brainstorming activities
• Collective edit inference by the cycles of feedback
• Reflective thinking on metacognition using prompts and self-assessment
• Provide collaboration within the cohort be facilitated by jointly engaging AI tools

**4.3.2 Behavioral intention for continued cognitive partnership**

**Student Voice - Sustained Engagement:** “ *These tools are useful, but I do not think we need to rely on them fully. They ought to be wisely exploited. I will use them again in my next courses because I think better not less with them.”*

This reaction is evidence of high levels of manipulation of cognitive partnership concepts, meaning that students gained the analytical awareness required to commit to persistent ethical AI collaboration under active learning conditions.

The impressive behavioral intention rates (86% expressed intentions to continue using AI), along with the indicators of the critical awareness, allows supposing that the active learning model has been effective in terms of supporting responsible adoption of technology instead of mindless dependence.

### 4.4 Active learning process indicators

The qualitative analysis demonstrated particular signs of successful development of the effective processes of active learning in the cognitive partnership with AI-based tools:

**4.4.1 Experiential learning cycle evidence**

**Direct Experience:** AI drafting, revision, and multi-modal composition were consistently dealt with by students in a hands-on way. The discussions during the week demonstrated an increasingly informed analysis of AI cooperation strategies

**Abstract Conceptualization:** Increased writing principles and ethical considerations were evidenced by the students as they explained such principles

**Active Experimentation:** This is where students were observed testing out various strategies and perfecting their cognitive partnership strategies

**4.4.2 Collaborative learning enhancement**

Students indicated more peer interactions and collaborative problem-solving, which was supported by common AI tools and the reflection activities. The cognitive partnership model promoted rather than inhibited human-to-human collaboration, as students shared strategies, discussed ethical considerations, and engaged in collective reflection on AI integration experiences.

**4.4.3 Higher-order thinking development**

Observation of weekly discussions and analysis of interview transcripts revealed evidence of critical evaluation, synthesis, and creative application—higher-order thinking skills that characterize effective active learning environments. Students moved beyond surface-level AI interaction to engage in sophisticated analysis of AI outputs, evaluation of ethical considerations, and creative integration of AI suggestions within original compositions.

## 5. Discussion

### 5.1 AI-mediated active learning: Theoretical validation and performance enhancement

The substantial improvement in the experimental group (Z = -6.325, p < .001) indicates that AI tools facilitated the experiential learning cycle proposed by Kolb (1984) in EFL/ESL writing. Although the quasi-experimental design restricts causal inference, baseline equivalence, progressive patterns, interaction effects, and qualitative triangulation can be used to attribute differences to the pedagogical approach. The progressive improvement, which is observed since Test 5 (p = .001) is consistent with what Kolb proposed that learning will only intensify under repetition of experience, reflection, conceptualization, and experimentation. This builds the meta-analysis by Freeman et al. (2014) that has found the benefits of active learning in all disciplines, verifying its concepts in AI-based EFL settings. The 6-8 weeks development time implies that cognitive partnerships are to be maintained over time, which is consistent with recursive models of learning. The large effect sizes (r = 0.68 overall; r = 0.72 at Test 5) and Bonferroni-adjusted significance will remove any doubts about Type I error inflation and trivial effects. The less impressive results of the control group (Z = -2.128, p = 0.033) support the statement of Prince (2004) that passive instruction is not as effective. Importantly, this paper shows that AI can be used as a facilitator to active learning in the resource-scarce situations where a conventional implementation is restricted. It is supported by the interview evidence: The significance of Test 5-6 is consistent with the qualitative reports of partnership maturation and advanced AI evaluation strategies. Qualitative results describe the transformation of the attitude toward AI as an instrument to the attitude toward it as a partner that justifies the model of cognitive partnership by Luckin (2017). This is opposed to Chang et al. (2021) and Fitria (2021), in which AI was viewed as feedback. Ethical reflections and cycles seem to have made the application of AI a true partnership. Student comments such as I learned to synthesize its recommendations with my own thoughts make evidence of the stage of abstract conceptualization in Kolb. In contrast to the case reported by Yan (2023), who raised dependent issues, the ethical procedure in this case reduced risks at the expense of AI, maintaining its cognitive advantages.

Although the quantitative evidence proves beneficial performance, to learn processes that AI facilitated active learning, it is essential to consider the metacognitive processes disclosed in qualitative data.

### 5.2 Active learning processes: Evidence and theoretical alignment

Metacognitive awareness, reflected in comments like “ *I started to notice when I was depending too much on AI*,” validates Bonwell and Eison’s (1991) assertion that active learning requires reflection. This distinguishes the study from prior AI writing research. Liu et al. (2021) also noted metacognitive improvements but lacked the systematic ethical framework used here. Structured prompts ensured AI use remained cognitively demanding, aligning with Prince’s (2004) definition of meaningful learning. AI tools enhanced rather than inhibited collaboration. Students reported increased peer interaction through shared AI engagement and reflection tasks, extending Johnson and Johnson’s (2009) collaborative learning theory into AI-mediated contexts. This contrasts with Anderson and Rainie’s (2023) concerns about AI reducing collaboration, as the partnership model positioned AI as a facilitator rather than a replacement.

### 5.3 Gender-differentiated engagement: Theoretical implications

Female students showed 19% higher engagement and greater improvement (p < .001) than males (p = .010) in AI-mediated active learning. In contrast, in the control group, males improved significantly (p < .001) while females’ gains were insignificant (p = .062). This reversal suggests AI-mediated environments may align better with learning preferences associated with reflective and collaborative engagement.

Qualitative data point to stronger responsiveness among females to reflection and metacognition in Kolb’s cycle. This extends Al Mahmud’s (2023) findings by showing that pedagogical framing mediates gendered outcomes. These results support Vygotsky’s (1978) view that scaffolding must match learner characteristics. While both genders benefited, the framework appears particularly effective for those favoring reflective collaboration. The significant gains for males (p = .010) suggest the approach benefits all learners while amplifying certain preferences.

### 5.4 Resource constraint mitigation: Theoretical and practical significance

The AI tools were used to overcome the issue of high enrollment in EFL classes, which supported the idea of AI being an equity tool mentioned by Bliksten (2016). Access to individualized instruction was democratized through reports of getting personalized feedback whenever they needed it through cognitive partnerships. This expands Popenici and Kerr (2017) and Renz et al. (2020) and offers solid empirical support that AI could be used to address the obstacles to active learning. The model overcame the conflict between large classes and individual feedback, which is a barrier to active pedagogy traditionally. The findings can substantiate the perspective of Godwin-Jones (2019), according to which technology can facilitate pedagogy but cannot substitute it, as it allows active learning to take place under limitations. According to critics, technology is not the solution to pedagogical problems, but this paper demonstrates that even in challenging situations, AI that is incorporated into grounded structures can support student-centered practices.

### 5.5 Technology acceptance and active learning integration

The Technology Acceptance Model (TAM) is confirmed by high-perceived usefulness (79.7%) and intention to continue use (86%). Notably, by engaging in pedagogical integration, but not AI as an isolated tool, acceptance was enhanced. Such responses by students as they make me think better than think less are critical evaluations and not passive adoptions and represent the issue that Davis (1989) discusses about uncritical acceptance. The protocol (See Appendix 7) served as a non-compliance governance framework. The alignment of EU AI Act (transparency through disclosure), ( European Commission, 2024), UNESCO principles (critical literacy) ( UNESCO, 2023) and Safe AI standards (dependency prevention) the international frameworks were converted into the classroom practice. Mitigation Risk register mitigation in the case of dishonesty, dependency, bias can be seen as a demonstration of mitigation in a scalable way. Ethics awareness (M = 3.4/4.0) is high, and 91 per cent disclosure confirms effectiveness.

All these ethical, pedagogical, and practical aspects are important to inform larger theoretical synthesis of AI-mediated active learning.

### 5.6 Theoretical synthesis: Active learning–GenAI synergy framework

Findings validate the Active Learning–GenAI Synergy Framework, showing AI can function as cognitive partners, metacognitive mirrors, and equity tools. This extends theories of AI in education by embedding technology within active learning, rather than as separate interventions. The achievement of the framework concerning the propensity to write and the acquisition of digital literacies endorses the vision of cognitive partnerships proposed by Holmes et al. (2019) as a tangible illustration of resource-limited situations. It was established by carrying out an implementation using ethical procedures and experiential learning processes. AI reduced cognitive load while enhancing higher-order thinking, challenging assumptions that assistance undermines cognition. This suggests AI-mediated learning can both reduce routine burdens and increase complex engagement. The finding extends Gayed et al.’s (2022) work on hybrid systems, showing cognitive partnerships simultaneously lighten basic demands while fostering deeper processes. The framework achieved this duality by structuring AI use around progressive experiential cycles.

### 5.7 Implications for EFL writing pedagogy

The shift from product- to process-focused instruction validates Flower and Hayes’s (1981) theory while extending it into AI-mediated contexts, supporting Graham and Perin’s (2007) process approach, highlighting AI-enabled individualized scaffolding in large classes, sustaining agency and critical thought. Improvements in coherence, structure, and revision directly address Pakistani EFL/ESL challenges noted by Mahmood et al. (2020). Multimodal integration expanded academic writing to include digital composition, offering practical strategies for contemporary literacy in constrained settings.

### 5.8 Unexpected findings and theoretical implications

High engagement combined with critical awareness challenges dependency concerns, showing students can maintain an authorial voice while using AI. This supports the concept of “critical dependence”, where learners leverage AI while retaining evaluation and agency. The observed 6–8 week maturation of cognitive partnerships highlights sustained engagement needs, countering assumptions of immediate technological benefit while validating Kolb’s recursive model. Progressive improvement suggests long-term integration is preferable to short-term adoption, offering guidance for sustainable AI pedagogy.

### 5.9 Limitations and future directions

Three major categories of limitation arise.

**Methodological:** The quasi-experimental design in intact classes does not allow causal inference, although baseline equivalence was established and data triangulation was employed to strengthen internal validity.

**Contextual:** Higher education context peculiarities of Pakistan, specific AI tools and 15 weeks of interest limits the generalizability.

**Measurement:** Ethical awareness, which is prone to social desirability, is not tested through long-term retention.

The future research needs to focus on the following aspects, including randomized controlled trials, cross-cultural validation, longitudinal tracking, and systematic comparison of AI tools and pedagogical frameworks.

## 6. Conclusion

This preliminary study offers some initial evidence that resource-constrained contexts may see the use of ethically scaffolded AI tools to improve active learning in EFL/ESL writing. The results that were measured were: better writing performance (experimental r = 0.68 versus control r = 0.23), development of ethical awareness (M = 1.8 → 3.4), and high levels of technology acceptance (86% intention to continue use). The confounds at the programme level (intact classes across various degree programs) and the specific nature of the context (Pakistani business students, ChatGPT. Claude and Meta AI, 15-week intervention) restrict the transferability and causal inference. Most importantly, the study confirms that the educational outcomes depend on the pedagogical structure that is used to mediate the interaction with AI and not on the technology itself. This result has far-reaching implications on AI integration tactics, indicating that funding on pedagogical growth and business ethics is critical in achieving AI in education without undermining the student agency and critical reasoning necessary to achieve learning success in the 21 st century.

Most significantly, the research establishes that the pedagogical framework mediating AI interaction—rather than the technology itself—determines educational outcomes. This finding has profound implications for AI integration strategies, suggesting that investment in pedagogical development and ethical frameworks is crucial for realizing AI’s educational potential while maintaining the student agency and critical thinking essential for 21st-century learning success.

## AI usage statement

Artificial Intelligence (AI) tools were used to support the preparation of this manuscript in limited ways. Specifically, **Grammarly** was employed to refine grammar, spelling, punctuation, and style. At the same time, **ChatGPT (OpenAI 4.0) and Claude AI** were used to assist with language editing, formatting consistency, reference cross-checking, and ensuring accuracy in in-text citations and the end reference list. Neither tool was used to generate original content, research ideas, or data analysis. All substantive intellectual contributions, interpretations, and arguments presented in this manuscript are the sole work of the authors.

## Data Availability

The data is available in the Zenodo repository ( https://doi.org/10.5281/zenodo.18449575; Malik, 2026), anonymized datasets, interview transcripts, full interview protocol, ethical reflections prompts (Weeks 1-15), AI prompt templates, scoring rubrics with anchors, coding framework, and implementation guide. Any publicly available content can be copied. Data are available under the terms of the Creative Commons Attribution 4.0 International License (CC BY 4.0).

## References

[ref1] Al Mahmud F : Investigating EFL students’ writing skills through artificial intelligence: Wordtune application as a tool. *Journal of Language Teaching and Research.* 2023;14(5):1395–1404. 10.17507/jltr.1405.15

[ref2] Anderson J Rainie L : *The future of human agency: How artificial intelligence will impact learning and work.* Pew Research Center;2023. Reference Source

[ref3] Bereiter C Scardamalia M : *The psychology of written composition.* Lawrence Erlbaum Associates;1987.

[ref4] Blikstein P : Computationally enhanced constructionism: Contextualizing the new revolution in artificial intelligence in education. *J. Learn. Sci.* 2016;25(4):563–591. 10.1080/10508406.2016.1232279

[ref5] Bonwell CC Eison JA : *Active learning: Creating excitement in the classroom.* The George Washington University, School of Education and Human Development;1991. (ASHE-ERIC Higher Education Report No. 1).

[ref6] Braun V Clarke V : Using thematic analysis in psychology. *Qual. Res. Psychol.* 2006;3(2):77–101. 10.1191/1478088706qp063oa

[ref7] Chang TS Li Y Huang HW : Exploring EFL students’ writing performance and their acceptance of AI-based automated writing feedback. *Proceedings of the 2021 2nd International Conference on Education Development and Studies.* Association for Computing Machinery;2021; pp.31–35. 10.1145/3431782.3431787

[ref8] Chen L Chen P Lin Z : Artificial intelligence in education: A review. *IEEE Access.* 2020;8:75264–75278. 10.1109/ACCESS.2020.2988510

[ref9] Davis FD : Perceived usefulness, perceived ease of use, and user acceptance of information technology. *MIS Q.* 1989;13(3):319–340. 10.2307/249008

[ref45] European Commission : *EU AI Act: First regulation on artificial intelligence.* 2024. https://artificialintelligenceact.eu/

[ref10] Fitria TN : Artificial intelligence (AI) technology in OpenAI ChatGPT application: A review of ChatGPT in writing English essay. *ELT Forum: Journal of English Language Teaching.* 2021;12(1):44–58. 10.15294/elt.v12i1.5002

[ref11] Flower L Hayes JR : A cognitive process theory of writing. *Coll. Compos. Commun.* 1981;32(4):365–387. 10.2307/356600

[ref12] Freeman S Eddy SL McDonough M : Active learning increases student performance in science, engineering, and mathematics. *Proc. Natl. Acad. Sci.* 2014;111(23):8410–8415. 10.1073/pnas.1319030111 24821756 PMC4060654

[ref13] Gayed JM Carlon MKJ Oriola AM : Exploring an AI-based writing assistant’s impact on English language learners. *Computers & Education: Artificial Intelligence.* 2022;3:100055. 10.1016/j.caeai.2022.100055

[ref14] Godwin-Jones R : Artificial intelligence and language learning: Opportunities and threats. *Lang. Learn. Technol.* 2019;5(3):7–12. 10125/44667. 10.64152/10125/44559

[ref15] Graham S Perin D : A meta-analysis of writing instruction for adolescent students. *J. Educ. Psychol.* 2007;99(3):445–476. 10.1037/0022-0663.99.3.445

[ref16] Higher Education Commission (HEC): *Draft policy framework for ethical use of Generative AI tools in Pakistani Higher Education Institutions (HEIs).* Islamabad, Pakistan: Higher Education Commission;2023.

[ref17] Holmes W Bialik M Fadel C : *Artificial intelligence in education: Promises and implications for teaching and learning.* Center for Curriculum Redesign;2019.

[ref18] Huang X Zou D Cheng G : Trends, research issues, and applications of artificial intelligence in language education. *Educ. Technol. Soc.* 2023;26(2):112–131. 10.30191/ETS.202304_26(2).0009

[ref19] Johnson DW Johnson RT : An educational psychology success story: Social interdependence theory and cooperative learning. *Educ. Res.* 2009;38(5):365–379. 10.3102/0013189X09339057

[ref20] Imran M Almusharraf N : Analyzing the role of ChatGPT as a writing assistant at higher education level: A systematic review of the literature. *Contemporary Educ. Technol.* 2023;15(4):ep464. 10.30935/cedtech/13605

[ref21] Imran M Almusharraf N : Google Gemini as a next generation AI educational tool: a review of emerging educational technology. *Smart Learning Environment.* 2024;11(1). 10.1186/s40561-024-00310-z

[ref22] Imran M Almusharraf N Ahmed S : Personalization of E-Learning: Future Trends, Opportunities, and Challenges. *International Journal of Interactive Mobile Technologies (iJIM).* 2024;18(10):4–18. 10.3991/ijim.v18i10.47053

[ref23] Kolb DA : *Experiential learning: Experience as the source of learning and development.* Prentice Hall;1984.

[ref24] Liu C Hou J Tu YF : Incorporating a reflective thinking promoting mechanism into artificial intelligence-supported English writing environments. *Interact. Learn. Environ.* 2021;31(6):3340–3359. 10.1080/10494820.2021.1927115

[ref25] Luckin R : Towards artificial intelligence-based assessment systems. *Nat. Hum. Behav.* 2017;1:0028. 10.1038/s41562-016-0028

[ref26] Mahmood R Khan NA Batool A : Challenges in academic English writing: A case of undergraduates in Pakistani higher education. *International Journal of English Linguistics.* 2020;10(6):85–92. 10.5539/ijel.v10n6p85

[ref43] Malik S : Dataset for Writing activities.[Data set]. *Zenodo.* 2025. 10.5281/zenodo.17417736

[ref27] McNiff J Whitehead J : *All you need to know about action research.* SAGE Publications; 2nd ed. 2011.

[ref28] Nazari N Shabbir MS Setiawan R : Application of artificial intelligence-powered digital writing assistant in higher education: Randomized controlled trial. *Heliyon.* 2021;7(5):e07014. 10.1016/j.heliyon.2021.e07014 34027198 PMC8131255

[ref29] Nobles S Paganucci L : Do Digital Writing Tools Deliver? Student Perceptions of Writing Quality Using Digital Tools and Online Writing Environments. *Computers and Composition: Part A.* 2015;38:16–31. 10.1016/j.compcom.2015.09.001

[ref31] Popenici SAD Kerr S : Exploring the impact of artificial intelligence on teaching and learning in higher education. *Res. Pract. Technol. Enhanc. Learn.* 2017;12(1):22. 10.1186/s41039-017-0062-8 30595727 PMC6294271

[ref32] Prince M : Does active learning work? A review of the research. *J. Eng. Educ.* 2004;93(3):223–231. 10.1002/j.2168-9830.2004.tb00809.x

[ref33] Purcell A Smith B Jones C : Enhancing student writing: Scaffolding and collaborative revision in digital platforms. *J. Educ. Technol.* 2013;10(2):123–135.

[ref46] Rashid S Malik S : Beyond Universal Models: Predicting Trait Emotional Intelligence’s Context-Contingent Effects on EFL Learners’ Attitudes, Motivation, Anxiety, and Engagement. *Education Sciences.* 2025;15(9):1137. 10.3390/educsci15091137

[ref34] Renz A Krishnaraja S Gronau N : Demystifying artificial intelligence in education: How AI can support personalized and adaptive learning. *Int. J. Educ. Technol. High. Educ.* 2020;17(1):1–16. 10.1186/s41239-020-00218-x

[ref35] Song C Song Y : Enhancing academic writing skills and motivation: Assessing the efficacy of ChatGPT in AI-assisted language learning for EFL students. *Front. Psychol.* 2023;14:1260843. 10.3389/fpsyg.2023.1260843 38162975 PMC10754989

[ref36] Stringer ET : *Action research.* SAGE Publications; 4th ed. 2014.

[ref37] Su Y Lin Y Lai C : Collaborating with ChatGPT in argumentative writing classrooms. *Assess. Writ.* 2023;57:100752. 10.1016/j.asw.2023.100752

[ref38] UNESCO: *AI and education: Guidance for policy-makers.* Scientific and Cultural Organization: United Nations Educational;2021. Reference Source

[ref49] UNESCO : *Guidance for generative AI in education and research.* 2023. https://www.unesco.org/en/articles/guidance-generative-ai-education-and-research

[ref39] Vygotsky LS : *Mind in society: The development of higher psychological processes.* Harvard University Press;1978.

[ref40] Yan Z : Student perceptions of the benefits and drawbacks of generative AI for academic writing tasks. *Smart Learning Environments.* 2023;10:53. 10.1186/s40561-023-00270-z

[ref41] Yang X Li H Zhang M : Interactive AI for student motivation and engagement in language learning. *Comput. Educ.* 2022;179:104411. 10.1016/j.compedu.2021.104411

[ref42] Zawacki-Richter O Marín VI Bond M : Systematic review of research on artificial intelligence applications in higher education—Where are the educators? *Int. J. Educ. Technol. High. Educ.* 2019;16(1):39. 10.1186/s41239-019-0171-0

