# Supplementary material for: The Active Learning–GenAI Synergy Framework: Ethical Integration of Generative AI in EFL/ESL Writing in Resource-Constrained Contexts
Source: F1000Res. 2026 Mar 3;14:1210. Originally published 2025 Nov 5. [Version 2] doi: 10.12688/f1000research.171435.2 (PMC12961280; doi:10.12688/f1000research.171435.2)
Supplement: Peer review history

# Reviewer report: Reviewer response for version 2

**DOI:** 10.5256/f1000research.196148.r464072

**Published:** 2026-03-04

**Authors:** Francisco José García-Peñalvo

**Affiliations:** 1 Universidad de Salamanca, Salamanca, Spain

The paper has been improved with the changes made.

 My only recommendation is to include a reference to Safe AI in the Education Manifesto and its principles, following the criteria used with UNESCO or Act AI.

 The Manifesto reference is:

 Alier, M., García-Peñalvo, F. J., Casañ, M. J., Pereira, J. A., & Llorens-Largo, F. (2024). *Safe AI in Education Manifesto. Version 0.4.0*. https://manifesto.safeaieducation.org.

 The principles behind the Manifesto are explained in:

 García-Peñalvo, F. J., Alier, M., Pereira, J. A., & Casañ, M. J. (2024). Safe, Transparent, and Ethical Artificial Intelligence: Keys to Quality Sustainable Education (SDG4). *IJERI – International Journal of Educational Research and Innovation*(22), 1–21. https://doi.org/10.46661/ijeri.11036

Is the work clearly and accurately presented and does it cite the current literature?

Partly

If applicable, is the statistical analysis and its interpretation appropriate?

Yes

Are all the source data underlying the results available to ensure full reproducibility?

Partly

Is the study design appropriate and is the work technically sound?

Partly

Are the conclusions drawn adequately supported by the results?

Partly

Are sufficient details of methods and analysis provided to allow replication by others?

No

Reviewer Expertise:

Artificial Intelligence in Education

I confirm that I have read this submission and believe that I have an appropriate level of expertise to confirm that it is of an acceptable scientific standard.

# Reviewer report: Reviewer response for version 1

**DOI:** 10.5256/f1000research.189043.r443883

**Published:** 2026-01-06

**Authors:** Francisco José García-Peñalvo

**Affiliations:** 1 Universidad de Salamanca, Salamanca, Spain

This mixed-methods action research examines the effectiveness of an “Active Learning–GenAI Synergy Framework” for EFL/ESL writing. Intact classes in Pakistan receive AI-mediated active learning or traditional instruction for 15 weeks. Pre- and post-tests are analysed with non-parametric statistics, and eight interviews explore student experiences and ethical awareness. The authors report larger writing gains and more positive perceptions in the AI group.

 General comments

 The manuscript presents a precise aim and an accessible narrative; however, clarity is reduced by fragmented sectioning and repeated “mini-sections.” More importantly, the ethical discussion and governance context do not engage with major, current reference points for GenAI in education, particularly the EU AI Act’s risk/transparency framing, UNESCO’s guidance for generative AI in teaching/research, and the Safe AI in Education Manifesto.

 A pragmatic mixed-methods, classroom-based design is appropriate for early evidence. However, the core comparison is confounded because experimental and control groups are intact classes from different programmes (i.e., not only “AI vs no AI”). This threatens internal validity and requires either (a) stronger controls (matching, covariates, teacher/time controls) or (b) reframing claims as exploratory and context-bound.

 Key replication materials are missing or under-specified, including the exact AI tools and versions, prompt templates, permitted/forbidden uses, the workflow for “ethical reflection prompts”, and detailed scoring/rubric procedures (including rater training and reliability). Without these, other educators cannot reproduce the intervention with fidelity, and readers cannot judge whether gains come from pedagogy, tool choice, instructor effects, or assessment practices.

 The choice of non-parametric tests can be reasonable for writing scores. Still, the reporting is incomplete: “effect size” is described qualitatively (e.g., “large”) without a defined metric, and repeated intermediate testing raises concerns about multiple comparisons. Interpretation sometimes implies causality and broad generalisability despite design confounds.

 The Zenodo record represents a significant step toward openness, featuring quantitative datasets (e.g., SPSS/XLSX) and supporting materials.

 The conclusions are directionally aligned with the reported gains, but currently overstate certainty. They should be tightened to reflect what was actually measured (writing performance and perceptions from a small qualitative sample), and should explicitly acknowledge the programme-level confound and contextual limits.

 Specific comments

 Structure, language (English) quality

 The manuscript is readable, but it requires substantial editing. Many very short sections and sections with only one subsection disrupt the narrative. Consolidate headings, reduce repetition, and ensure each section has a clear purpose and transition. English requires careful proofreading (including grammar, tense consistency, and academic tone).

 Methodology

 The mixed-methods, quasi-experimental pre/post design is reasonable for classroom settings; however, the comparison is confounded because the experimental and control groups are intact classes from different degree programs. Authors should report baseline equivalence (pre-test distributions, prior English proficiency, teacher effects) and justify assignment procedures. Describe the intervention with enough detail to replicate: exact AI tools, versions, access conditions, prompt templates, guardrails, and the weekly active-learning sequence. Provide the writing rubric (criteria, levels), scoring procedure, rater training, inter-rater reliability, and how missing data were handled. Operationalise and measure’ ethical awareness’ beyond reflection prompts. Clarify interview sampling, protocol, transcription, and coding reliability (e.g., double-coding and agreement) independently.

 Results and discussion

 Results suggest improved writing performance for the AI-active learning class, with progressive gains across the term, and interview themes consistent with perceived usefulness and metacognitive support. However, effect sizes are reported qualitatively (“large”) without a straightforward metric, and multiple comparisons across intermediate tests need control or justification. The discussion sometimes treats the findings as causal and broadly generalisable, despite programme-level confounds and a small qualitative sample (n=8). Strengthen triangulation by linking specific qualitative codes to quantitative patterns and by discussing alternative explanations, bias, and context limits in resource-constrained education settings.

 Conclusions

 Conclusions are promising but overstated. Reframe claims as exploratory, align them with measured outcomes, and explicitly acknowledge confounds and limited transferability to other institutions.

 References (quality, completeness, and consistency)

 Current literature coverage: Update the ethics/governance coverage to engage with explicitly:

- EU AI Act (Regulation (EU) 2024/1689)—risk-based obligations, transparency expectations, and governance concepts relevant to educational deployments.
- UNESCO guidance for generative AI in education and research—human agency, inclusion, privacy, institutional readiness, and responsible use.
- Safe AI in Education Manifesto - https://manifesto.safeaieducation.org (and associated scholarly discussion, DOI: 10.46661/ijeri.11036).

Please run a strict audit to ensure (1) every in-text citation appears in the reference list with complete metadata, and (2) every reference is cited in the text (remove uncited items). This is especially important after adding the missing governance frameworks.

 Ethical and governance critique

 The manuscript’s ethics content is primarily procedural (approval and consent, along with brief guidance) and does not yet serve as a governance argument for GenAI in education.

 To strengthen:

 1. Map your “ethical integration protocol” to established frameworks:

 -EU AI Act: explicitly discuss transparency to learners, documentation of system limits, and risk management concepts that readers can operationalise.

 - UNESCO guidance: align reflection prompts and classroom rules with UNESCO’s recommended institutional and pedagogical safeguards.

 - Safe AI in Education Manifesto: show how your protocol supports principles such as human oversight, privacy, pedagogical alignment, and transparency.

 2. Define what you mean by “ethical awareness” and how it is assessed (observable indicators, rubric, or validated instrument), rather than describing reflection prompts only.

 3. Add a short risk register (common GenAI risks in writing: hallucinations, bias, privacy leakage, overreliance, academic integrity ambiguity) and show how each is mitigated and monitored during the intervention.

 Mistakes and inconsistencies

- Fragmented structure: many numbered sub-sections are extremely short and disrupt reading flow; some sections have only a single subsection (suggesting restructuring is needed).
- Effect size reporting is not transparent: “Large” is not a statistic; specify the effect size metric (e.g., r, Cliff’s delta) and how it was computed for each comparison.
- Causal language risk: parts of the discussion/conclusion imply the framework caused gains, but the design (intact classes from different programmes) cannot fully support that inference.
- Ethics section lacks anchoring: ethics claims are not situated in primary education-focused GenAI governance references (AI Act / UNESCO / Safe AI Manifesto).
- Replication gap: intervention materials (prompts, tool settings, permitted/forbidden uses, marking rubric, rater procedure) are not sufficiently disclosed for others to replicate.

Areas of improvement

 Prioritise scientific clarity and governance. (

- Redesign the manuscript structure: merge micro-sections, add a clear conceptual diagram of the framework, and provide a concise narrative linking RQs, intervention elements, measures, and findings.
- Strengthen ethics by mapping your protocol to widely used frameworks: the EU AI Act’s transparency and risk-management obligations for AI systems, UNESCO’s guidance for GenAI in education and research, and the Safe AI in Education Manifesto’s principles (human oversight, confidentiality, alignment with pedagogy, accuracy, and transparency).
- Improve methodological rigour: report baseline equivalence, add sensitivity analyses, and temper causal language.
- Provide replication materials (rubrics, prompts, training slides, reflection prompts, and coding scheme) and data documentation.
- Expand limitations/future work into concrete next steps (randomised or matched designs, multi-site studies, and learning analytics of actual AI use), and cost-benefit considerations for instructors.

Final decision

 Approved with Reservations. The topic is timely, and the evidence is encouraging; however, the quasi-experimental comparison is confounded, replication details are insufficient, and the ethics discussion requires alignment with major governance frameworks.

Is the work clearly and accurately presented and does it cite the current literature?

Partly

If applicable, is the statistical analysis and its interpretation appropriate?

Yes

Are all the source data underlying the results available to ensure full reproducibility?

Partly

Is the study design appropriate and is the work technically sound?

Partly

Are the conclusions drawn adequately supported by the results?

Partly

Are sufficient details of methods and analysis provided to allow replication by others?

No

Reviewer Expertise:

Artificial Intelligence in Education

I confirm that I have read this submission and believe that I have an appropriate level of expertise to confirm that it is of an acceptable scientific standard, however I have significant reservations, as outlined above.

# Response

**Published:** 2026-02-03

**Authors:** Sadia Malik

**Affiliations:** English, Bahauddin Zakariya University, Multan, Punjab, Pakistan

1. **Reviewer Comment:** The ethical and governance discussion does not engage with major, current GenAI frameworks, particularly the EU AI Act, UNESCO guidance for generative AI in education and research, and the Safe AI in Education Manifesto.

- **Response:** This limitation is acknowledged, and Section 2.5 has been strengthened by incorporating discussion of the EU AI Act (2024), UNESCO's guidance on generative AI in education and research (2023), and Safe AI frameworks, demonstrating how the independently developed ethical protocol converges with these international standards while providing practical implementation strategies for resource-constrained contexts where such frameworks offer limited operational guidance. (See p. 6, Section 2.5; p. 9, Section 3.5; p. 19, Section 5.5)

2. **Reviewer Comment:** Although the mixed-methods, quasi-experimental pre-/post-test design is appropriate for classroom research, the core comparison is confounded because experimental and control groups are intact classes from different degree programmes, threatening internal validity and requiring stronger controls or reframing of claims as exploratory.

- **Response:** This is a valid methodological concern regarding the quasi-experimental design. The reviewer correctly identifies that using intact classes from different degree programs (Digital Marketing vs. BBA) introduces potential confounding variables that could threaten internal validity. The claims are reframed as exploratory. (See p. 8, Section 3.3)

3. **Reviewer Comment:** Baseline equivalence between groups is insufficiently reported, including pre-test distributions, prior English proficiency, and possible teacher effects. 

- **Response: **Baseline equivalence reporting has been strengthened. Pre-test analysis confirmed no significant differences in writing performance (U = 2,561, p = .532), prior English proficiency (t = 0.58, p = .564), gender distribution (χ² = 1.89, p = .169), or age (t = 0.73, p = .468). Both groups had equivalent admission requirements (IELTS 5.5) and qualified instructors. Section 3.3 now includes comprehensive baseline data with score distributions and demographic characteristics.  (See pp. 8-9, Section 3.3)

4. **Reviewer Comment: **Key replication details are missing or under-specified, including exact AI tools and versions, access conditions, prompt templates, permitted and forbidden uses, ethical reflection workflows, and the weekly active-learning sequence.

- **Response: **Replication transparency was enhanced in Sections 3.3 and 3.5 through documentation of participant characteristics, instructional conditions, ethical integration protocols, and the AI tools used (ChatGPT, Claude AI, Meta AI, Canva). Although full prompt templates are provided through the Zenodo repository and in the appendices, guided instructional procedures and ethical usage principles are clearly described, supporting pedagogical transferability in comparable ESL contexts. (See p. 8, Section 3.3; p. 9, Section 3.4; p. 10, Section 3.5)

5. **Reviewer Comment: **Writing assessment procedures lack sufficient detail, including rubric criteria and levels, scoring procedures, rater training, inter-rater reliability, and handling of missing data; additionally, “ethical awareness” is not clearly operationalised or measured beyond reflection prompts.

- **Response: **Section 3.6 now details assessment procedures: 100-point analytic rubric (coherence, organization, grammar, critical thinking—25% each), two trained raters, inter-rater reliability κ = 0.84, third-rater adjudication for discrepancies. Ethical awareness operationalized through coded reflection quality (4-point scale), AI disclosure accuracy, and interview analysis, showing progression from M=1.8 to M=3.4 across 15 weeks. (See p. 11, Section 3.6)

6. **Reviewer Comment: **The qualitative methodology requires clearer reporting of interview sampling, interview protocol, transcription process, coding procedures, and coding reliability.

- **Response: **Qualitative methodology is detailed in Sections 3.6 and 3.8. Purposive sampling selected eight participants (diverse performance levels, gender balanced). Semi-structured interviews (30-45 min) explored five domains; audio-recorded, transcribed verbatim, and accuracy-checked. Braun and Clarke's (2006) six-phase thematic analysis was applied; two researchers independently coded 25% (κ = 0.82). Interview protocol added to Appendix 7. (See p. 12, Section 3.6; p. 12, Section 3.8)

7. **Reviewer Comment: **The use of non-parametric tests is reasonable; however, statistical reporting is incomplete, as effect sizes are described qualitatively without specifying metrics, and repeated intermediate testing raises concerns about multiple comparisons without clear justification or control.

- **Response: **Effect sizes are now reported using rank-biserial correlation (r): experimental r = 0.68 (large), control r = 0.23 (small). Intermediate tests served as developmental tracking; Bonferroni correction (α = .008) was applied retrospectively—only Tests 5-6 retained significance (r = 0.72, r = 0.58), confirming that the findings are robust and not inflated by multiple comparisons. All tables updated with effect size metrics.  (See Tables 6-9, pp. 13-14 Sections 4.1.1-4.1.3)

8. **Reviewer Comment: **The discussion sometimes implies causality and broad generalisability despite design confounds; alternative explanations, contextual limitations, and stronger triangulation between quantitative results and qualitative findings are needed. 

- **Response: **Discussion revised to eliminate causal language ("suggests," "associated with" replacing "demonstrates," "proves"). Alternative explanations related to programme-level confounds and intact class design have been explicitly acknowledged in the manuscript. Contextual limitations explicitly stated (Pakistani context, specific tools, 15-week timeframe). Triangulation is strengthened by linking statistical patterns to qualitative evidence. Findings repositioned as context-specific, requiring replication. (See pp. 17-18, Section 5.1; p. 20, Section 5.9; p. 20, Section 6)

9. **Reviewer Comment: **The ethics section is largely procedural and does not function as a governance argument; the ethical integration protocol is not mapped to established frameworks (EU AI Act, UNESCO guidance, Safe AI in Education Manifesto), “ethical awareness” is not clearly defined, and no explicit GenAI risk register or mitigation strategy is provided.

- **Response: **Ethical governance is strengthened through alignment with the EU AI Act, UNESCO guidance, and Safe AI principles, emphasizing transparency, learner agency, critical literacy, and academic integrity (Section 2.5). These principles are operationalized in Sections 3.5–3.6 via guided instruction and integrity safeguards. Although no formal risk register is included, key risks (dependency, bias, dishonesty, privacy, and access) are mitigated through pedagogical controls, framing governance as integrated instructional practice rather than procedural compliance. (See p. 6, Section 2.5; pp. 10-11, Section 3.5-3.6; p. 19, Section 5.5)

**10.** **Reviewer Comment: **Conclusions are directionally aligned with results but overstate certainty; they should be reframed as exploratory, aligned with measured outcomes only, and explicitly acknowledge programme-level confounds and limited transferability.

- **Response: **The Conclusion was revised to emphasize the study’s exploratory nature, restrict claims to measured outcomes (e.g., r = 0.68; ethical awareness from M = 1.8 to M = 3.4), and acknowledge contextual constraints (Pakistani setting, specific AI tools, 15-week duration). Language is moderated, and findings are framed as preliminary, requiring validation through randomized and cross-cultural studies. (See p. 20, Section 6)

11. **Reviewer Comment: **Ethics and governance literature coverage is incomplete; key frameworks (EU AI Act, UNESCO guidance, Safe AI in Education Manifesto) are missing, and a full audit is required to ensure consistency between in-text citations and the reference list. 

- **Response: **Ethics and governance literature was expanded in Section 2.5 to incorporate the EU AI Act (2024), UNESCO guidance on generative AI in education and research (2023), and Safe AI principles. These frameworks are operationalized through the study’s ethical integration protocols detailed in Sections 3.5 and 3.7. In-text citations and references were reviewed to ensure consistency. One relevant reference is added at n. 34 (See p. 6, Section 2.5; p. 10, Section 3.5; pp. 21-24, References)

**12. Reviewer Comment: **The Zenodo record is a positive step toward openness, but it does not fully compensate for missing replication and documentation details.

- **Response: **A Zenodo repository is referenced in the Declaration section, providing access to replication materials, including interview protocols, ethical reflection prompts, AI task prompts, scoring rubrics, anonymized datasets, and coding frameworks. The manuscript directs readers to this repository while core methodological procedures are described, ensuring transparency due to the article's word limit/space constraints. However, we have added the same at the end of the article as appendices, in case the reviewer wants them included. (See p. 11, Section 3.6; p. 21, Availability of data and materials; pp. 24-29, Appendices)

13. **Reviewer Comment: **The study is timely and promising but approved with reservations due to confounded design, insufficient replication detail, and inadequate ethics and governance alignment.

- **Response: **Reviewer concerns were addressed through strengthened baseline equivalence reporting (p = .532), moderated interpretive language, and inclusion of effect sizes (experimental r = 0.68; control r = 0.23) with Bonferroni adjustment. Assessment procedures were clarified (analytic rubrics, inter-rater reliability κ = 0.84, and ethical awareness operationalization). Ethical governance aligns with EU AI Act, UNESCO, and Safe AI frameworks. Quantitative–qualitative triangulation was enhanced, conclusions reframed as exploratory, and replication materials shared via Zenodo and as appendices.

# Reviewer report: Reviewer response for version 1

**DOI:** 10.5256/f1000research.189043.r435292

**Published:** 2025-12-26

**Authors:** Laura Trujillo Liñán

**Affiliations:** 1 Universidad Panamericana, MÉXICO, CDMX, Mexico

The article is interesting for educators as it integrates a new way of teaching through the use of AI within an ethical framework. The results indicate the possibility of replicating this approach at other levels and in other institutions; likewise, it demonstrates that it is possible to educate successfully using AI-based resources.

Is the work clearly and accurately presented and does it cite the current literature?

Yes

If applicable, is the statistical analysis and its interpretation appropriate?

Yes

Are all the source data underlying the results available to ensure full reproducibility?

Partly

Is the study design appropriate and is the work technically sound?

Yes

Are the conclusions drawn adequately supported by the results?

Yes

Are sufficient details of methods and analysis provided to allow replication by others?

Yes

Reviewer Expertise:

Ethics, Philosophy, Metaphysics, Media, AI, Algorithms, Ethical Frameworks for AI, Philosophy of Technology.

I confirm that I have read this submission and believe that I have an appropriate level of expertise to confirm that it is of an acceptable scientific standard.

## References

[1] : Ethical and regulatory challenges of Generative AI in education: a systematic review. *Frontiers in Education* .2025;10: 10.3389/feduc.2025.1565938 10.3389/feduc.2025.1565938

# Response

**Published:** 2026-02-03

**Authors:** Sadia Malik

**Affiliations:** English, Bahauddin Zakariya University, Multan, Punjab, Pakistan

We are thankful to the reviewer for approving our article.
